# CBD lengthens sleep but shortens ripples and leads to intact simple but worse cumulative memory

**DOI:** 10.1016/j.isci.2023.108327

**Published:** 2023-10-24

**Authors:** Anumita Samanta, Adrian Aleman-Zapata, Kopal Agarwal, Pelin Özsezer, Alejandra Alonso, Jacqueline van der Meij, Abdelrahman Rayan, Irene Navarro-Lobato, Lisa Genzel

**Affiliations:** 1 Donders Institute for Brain, Cognition and Behaviour, Radboud University, Postbus 9010, 6500 GL, Nijmegen

**Keywords:** Pharmacology, Cognitive neuroscience

## Abstract

Cannabidiol (CBD) is on the rise as over-the-counter medication to treat sleep disturbances, anxiety, pain, and epilepsy due to its action on the excitatory/inhibitory balance in the brain. However, it remains unclear if CBD also leads to adverse effects on memory via changes of sleep macro- and microarchitecture. To investigate the effect of CBD on sleep and memory consolidation, we performed two experiments using the object space task testing for both simple and cumulative memory in rats. We show that oral CBD administration extended the sleep period but changed the properties of rest and non-REM sleep oscillations (delta, spindle, ripples). Specifically, CBD also led to less long (>100 ms) ripples and, consequently, worse cumulative memory consolidation. In contrast, simple memories were not affected. In sum, we can confirm the beneficial effect of CBD on sleep; however, this comes with changes in oscillations that negatively impact memory consolidation.

## Highlights

• CBD lengthens non-REM sleep
• CBD shortens ripples
• CBD negatively impacts cumulative memory performance
• CBD leaves simple memory intact

## Graphical abstract

**Figure:**
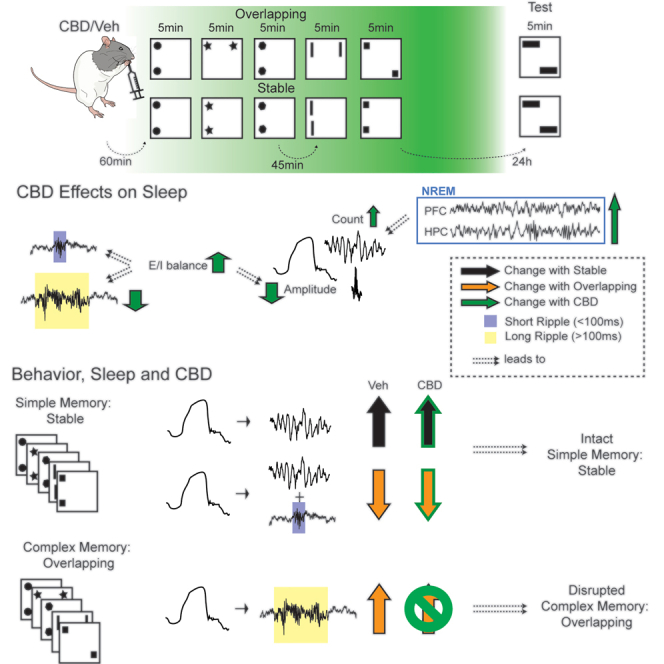

## Introduction

Cannabidiol (CBD) has become a popular remedy for many ailments and shows promising effects treating chronic pain, anxiety, and sleep disturbances.^1^^,^^2^ Furthermore, due to the action on inhibition in the brain, CBD is potentially a novel therapeutic agent for restoring excitation/inhibition balance in models of epilepsy^3^ and autism, which stem from dysregulation of inhibitory networks.^4^ CBD as well as CB1-receptor agonist have been shown to promote sleep in multiple pre-clinical and clinical studies.^5^^,^^6^^,^^7^^,^^8^^,^^9^^,^^10^ For example, in patients with neuropsychiatric disorders, CBD administration improved sleep quality in more than half of the patients up to a month after administration and alleviated anxiety symptoms.^10^ Interestingly, the effect of CBD on sleep is dose-dependent with high doses increasing sleep duration^11^ and the opposite effect is observed with low doses.^12^ CBD may also have different effects in the aged population in contrast to young adults and adolescents.^13^ Despite the rising use of CBD by various patients as an over-the-counter remedy, very little is known about how CBD affects the microstructure and memory functions of sleep, which could lead to side effects as seen with other sleep-modulating medications.^14^^,^^15^^,^^16^

Sleep is thought to play a critical role in the consolidation of memories, both for simple memories – such as the association of two items – as well as the more complex extraction of regularities across different experiences. Non-rapid eye movement (NonREM) sleep has been proposed as a critical stage in offline memory processing.^17^^,^^18^ The reactivation of cells active during encoding has been shown to occur during quiet wake and NonREM sleep and is potentially crucial to memory consolidation.^19^^,^^20^^,^^21^^,^^22^ These reactivation events occur during hippocampal high frequency burst oscillations (100–250 Hz) referred to as ripples.^23^ Interestingly, it had been shown that administration of CB1-agonist disrupts ripples in the hippocampus.^24^^,^^25^^,^^26^^,^^27^ However, to be able to consolidate memories during sleep, a coordinated action between the hippocampus and the neocortex around the ripples is needed. Two major neocortical events – delta waves (0.5–4 Hz) and spindles (9–20 Hz) – can occur in close coordination with the ripple, and this crosstalk between brain regions is crucial for offline memory processing.^28^^,^^29^^,^^30^^,^^31^ The characteristics and coordination of these microstructural sleep events are influenced by the depth of sleep as well as most sleep-inducing medications^15^^,^^16^; thus, there are likely effects of CBD on spindles and delta waves similar to the effect on ripples that could negatively impact their function in memory consolidation.

CBD can act as an antagonist of the CB1-receptors.^32^^,^^33^^,^^34^ These receptors are present on hippocampal cholecystokinin (CCK) and parvalbumin (PV) positive interneurons.^35^ CB1-receptors are found in the presynaptic axon terminals of the GABAergic interneurons, most prominently in the CA1-CA3 subfields.^36^^,^^37^ They act like retrograde messengers, wherein their activation in the presynaptic targets is triggered by depolarization in the post-synaptic targets.^35^ CB1-receptors potentially regulate the suppression of GABA-mediated transmission following the depolarization of hippocampal pyramidal neurons,^38^^,^^39^ which is a key player in the occurrence of ripples. The ripple component is initiated by the activation of pyramidal neurons in the CA1, in response to input from CA3^23^^,^^40^^,^^41^; however, the oscillation is maintained by interneuron activity.^42^ Thus, changes in ripples after CBD administration could stem from the change in the E/I balance.

To test the effect of CBD on sleep and sleep-related memory consolidation, we performed two experiments using the object space task^43^ in young, adult rats. This task enables measuring both simple and cumulative memory, where the former should be more resistant, whereas the latter should be more sensitive to manipulation effects. In the first experiment, we tested the effect of acute, oral CBD administration – mimicking human CBD intake^44^ – before learning and consolidation on memory expression the next day. For the second experiment, we conducted in fewer animals’ recordings of natural sleep and sleep oscillations after learning with electrophysiological implants targeting the hippocampus and the prefrontal cortex to elucidate how CBD affects the sleep macro- and microstructure. Finally, we returned to the animals from the behavioral experiment and recorded sleep-like states in anesthesia to confirm findings from the second experiment. Oral administration of CBD increased NonREM sleep at the end of the sleep period. Furthermore, it changed sleep microstructure – leading to less long ripples – that resulted in worse cumulative but intact simple memories tested the following day.

## Results

### CBD decreases cumulative memory expression the next day but leaves simple memory intact

**Figure 1:**
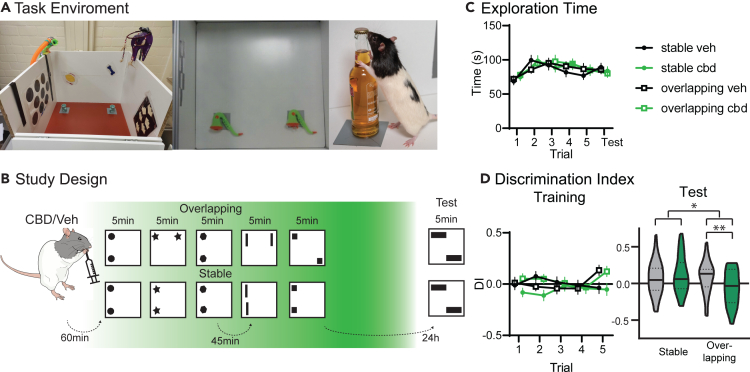
**Behavior** (A) Shown is the object exploration box (side view, top view and with animal). (B) Study Design. Rats received orally either vehicle or CBD (cross-over) and 60 min later were trained in the OS task with either Overlapping (one stable, one moving object location) or Stable (two stable object locations) condition (5-min trials, inter-trial interval 45 min) and tested 24 h later. Green shading in background indicates expected CBD concentration, since previous studies have shown CBD to reach maximum levels in the brain around 4–6 h after oral administration.^44^^,^^45^ (C) The exploration time remained stable for both conditions and treatment. (D) There was no difference in discrimination index (DI) during training for treatment, but as expected in the fifth trial both showed positive DIs in overlapping. At test, there was a significant condition × treatment interaction (p = 0.031, t-test overlapping Veh and CBD t_72_ = 2.74 p = 0.0078). ∗p < 0.05 n = 37.

Our first aim was to establish a behavioral effect of CBD administration targeting memory consolidation. For this, the object space task (Figure 1A) was combined with oral administration of CBD, in a similar dose as commonly used in oral administration to humans. Previous studies have shown oral administration of CBD to be effective in crossing the blood brain barrier and plasma concentrations are shown to reach their maximum around 4–6 h after oral administration.^44^^,^^45^Thus, CBD and vehicle-control was given 1 h before training start (1 h after light on, Figure 1B), such that the peak of CBD in the brain occurred after training during the main sleep period. Of note, all experiments were run counterbalanced and blinded. The experimenter was not aware of the current drug treatment of the rat.

Rats were trained in either the Stable or Overlapping condition, which test simple and cumulative memory respectively. In Stable, in each training trial identical object pairs (each trial different ones) are presented in the same two locations in the exploration box over 5 training trials. At the test (24 h after training), two new objects are again used and presented with one on a usual location but the other one placed in one of the other two corners. Neophilic animals will explore the object in the novel location more, independent of whether they just remembered the last experience or all five training trials (leading to positive Discrimination Index, DI). Thus, the Stable condition tests for simple location memory. In contrast, in Overlapping, already during training only one location remains stable, while the second object will be in one of the three other corners. Now, the test trial has the same configuration as the final training trial. Therefore, only if the rats created a cumulative memory, abstracted over multiple training trials, will they show a positive discrimination index at test. All animals underwent both conditions and both treatments, therefore four rounds of training-test, in a cross-over design. Treatment/condition sequences were counterbalanced across rats and object locations.

While CBD was administered before training, due to the slow uptake the main effect should be after training in the sleep period. In confirmation that CBD did not affect encoding behaviors, both exploration time (Figure 1C) and exploration preferences during training did not show a treatment effect (discrimination index, Figure 1D). As expected, in Overlapping a positive discrimination index was already seen in trial 5, due to the moving object-location during training. At test, there was a significant condition × treatment interaction (rmANOVA condition F_1,36_ = 5.6 p = 0.023, treatment F_1,36_ = 1.2 p = 0.28, interaction F_1,36_ = 5.1 p = 0.031 only including animals that showed memory in the vehicle condition. However, the same interaction effect was seen when all animals were included F_1,43_ = 3.0 p = 0.089). CBD administration led to disrupted cumulative memory expression (Overlapping condition, p < 0.01) but intact simple memory (Stable condition).

In sum, using a potentially translatable approach with oral administration of CBD, we showed a significant effect on behavior, where CBD led to intact simple but worse cumulative memory.

### CBD extends natural NonREM sleep

**Figure 2:**
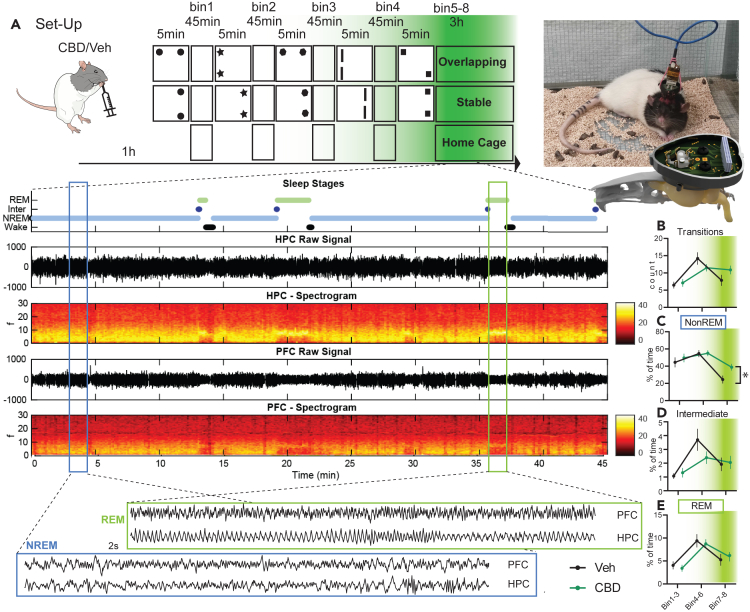
**Electrophysiological experiments** (A) Rats were implanted with a wire-drive (picture on the right). On study days they received CBD or Vehicle (Veh) orally and 1 h later started the OS training as in Figure 1 and spent the time between trials in the sleep recording box (4 × 45 min bin 1-4, 3 h after final trial bins 5–8). In addition to the conditions Overlapping and Stable, animals also spent one study day always in the Home Cage during the usual training periods (circadian, no-learning control). Below manually scored sleep stages as well as raw signal and spectrograms arising from the hippocampus (HPC) and prelimbic cortex (PFC) are shown for an example 45-min bin. Finally, for each NonREM (NREM) and REM state a 2 s example of raw LFP is presented. (B–E) Next separated for Bin 1-3, 4–6 and 7–8 the count of state-transition (B) as well as percent time spend in NonREM (C), Intermediate (D) and REM (E). Only NonREM showed a significant effect with more sleep in the final bins in rats treated with CBD (Transitions: time F_2,43_ = 17.2 p < 0.0001, treat F_1,22_ = 0.04 p = 0.8, interaction F_2,43_ = 3.7 p = 0.03; NonREM: time F_2,43_ = 17.3 p < 0.0001, treat F_1,22_ = 2.2 p = 0.15, interaction F_2,43_ = 1.5 p = 0.23; Intermediate time F_2,43_ = 12.5 p < 0.0001, treat F_1,22_ = 0.3 p = 0.6, interaction F_2,43_ = 2.6 p = 0.08; REM time F_2,43_ = 30.0 p < 0.0001, treat F_1,22_ = 0.04 p = 0.9, interaction F_2,43_ = 0.5 p = 0.6; effect size NonREM final bin 1.01). For (A–E) green shading implies expected effective CBD levels. Orthogonal comparisons ∗p < 0.05 n = 4.

Our second aim was to characterize the effects of CBD on sleep and sleep oscillations. For this, new rats (n = 4) were implanted with a wire-drive targeting the prelimbic cortex and hippocampus, which allowed us to acquire wake and sleep data in the task (5 times 5-min trials) and sleep box (4 × 45 min after trials 1–4 and for 3 h after trial 5, total 6 h) for each study day (study design Figure 2A, histology Figure S2). The recordings were divided into 5-min task and 45-min sleep-box bins (between each trial and 4 bins after the fifth trial). Raw signal and spectrogram for one such 45 min is presented in Figure 2A. The signal was manually scored for sleep stages NonREM, Intermediate and REM. Fitting to the idea that CBD promotes sleep, rats treated with CBD had more NonREM sleep but no changes in Intermediate and REM sleep nor the number of sleep stage transitions (Figures 2B–2E). However, the effect on NonREM sleep was modest; it only became apparent in the last two sleep bins, when control rats showed a decline in amount of NonREM sleep. Of note, this is also the time period where the maximum level of CBD should have reached the brain.

In sum, CBD led to more NonREM sleep at the end of the sleep period.

### CBD makes NonREM oscillations smaller

**Figure 3:**
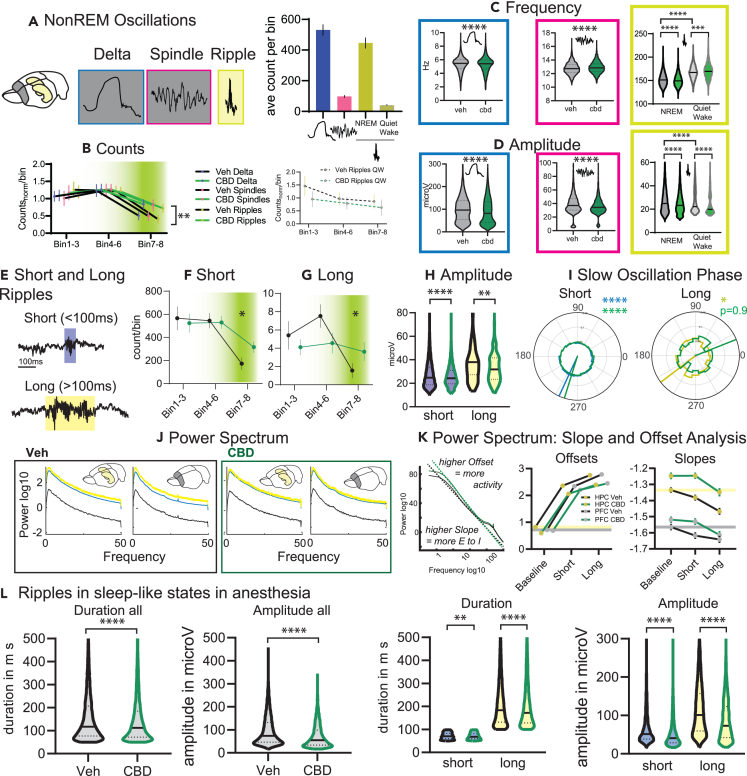
**NonREM Oscillations** (A) Cortical delta and spindles (both NonREM), and hippocampal ripples (NonREM and quiet wake in the sleep box) were detected. On the right, average counts per bin (types F_3,273_ = 121.6 p < 0.0001). (B) Counts for all types normalized by their average. There was a significant treatment and time interaction (treat F_1,30_ = 5.2 p = 0.029, bin F_2,40_ = 30.9 p < 0.001, treat X bin F_2, 60_ = 4.7 p = 0.012 all other p > 0.75). This was not seen for quiet wake ripples (p > 0.6). (C and D) From left to right for delta, spindle and ripple the intrinsic frequency (C) and amplitude (D) for all events (bin1-8). Delta and NonREM ripples were slower and smaller, spindles faster and smaller (all K-S D, p < 0.0001). Interestingly, quiet wake ripples were smaller but faster. (E) Examples of short and long ripples. (F and G) Counts for (F) short and (G) long ripples. (H) Amplitudes. (I) Slow oscillation phase, all short ripples were locked but for long ripples only in vehicles significant locking was seen. (J) Power spectrum for vehicle and CBD, always left for hippocampus and right for prelimbic cortex. Of note, in Veh there was more power for long than short ripples especially in the prelimbic cortex. This was not seen in CBD. (full model without baseline long/short F_1, 2792_ = 86.4 p < 0.001, HPC/PFC F_1, 2792_ = 302.9 p < 0.001, treat F_1, 2792_ = 31.8 p < 0.001, long/shortXHPC/PFC F_1, 2792_ = 8.2 p = 0.004, long/shortXtreat F_1, 2792_ = 11.1 p < 0.001 other interactions p > 0.5; only vehicle long/shortXHPC/PFC F_1, 1596_ = 7.3 p = 0.007 only CBD long/shortXHPC/PFC F_1, 1196_ = 2.1 p = 0.14). (K) Slope and Offset Analysis from left to right: how slopes and offsets are calculated, offset and slopes for ripple types and random selected baseline. Lines indicate vehicle baseline levels. Offsets: events F_2, 3588_ = 1592 p < 0.0001, treatment F_1, 3588_ = 63.15 p < 0.0001, events X treatment F_2, 3588_ = 4.5 p = 0.011, brain area X treatment F_1, 3588_ = 5.0 p = 0.026. Slopes: event F_2, 3588_ = 24.6 p < 0.0001, brain area F_1, 3588_ = 397.5 p < 0.0001, treatment F_1, 3588_ = 47.3 p < 0.0001, brain area X type F_1, 3588_ = 6.2 p = 0.0127 other p > 0.27). (L) Ripples detected in sleep-like states in anesthesia (n = 9 and 10 for Veh and CBD respectively). After CBD ripples were shorter and smaller and even after split into short and long ripples this effect remained the same for both types (Duration short K-S p = 0.0013 all other K-S D p < 0.0001), ∗∗p < 0.01, ∗∗∗∗p < 0.0001.

Next, we detected specific NonREM oscillations that are implied to be critical for memory consolidation.^18^ Delta and spindle oscillations in the prelimbic cortex and ripple oscillations in the hippocampal CA1 area were detected during NonREM sleep periods and in addition ripples during quiet wake in the sleep box. Deltas and NonREM ripples were much more numerous than spindles and quiet wake ripples (Figure 3A). To account for these differences, the counts of oscillations were normalized by their overall average and then, as for the sleep stages, separated for the different sleep bins. After CBD there were more deltas, spindles, and NonREM ripples in the final bins (Figure 3B); however, this was due to the increased amount of NonREM sleep, since there was no change in rates (Figure S3) and also no change in quiet wake ripples. CBD did change the properties of each type of oscillation. Deltas and NonREM ripples became slower (decreased intrinsic frequency), while spindles and quiet wake ripples became faster (Figure 3C). All four oscillations became smaller in amplitude (Figure 3D).

In sum, more NonREM sleep in CBD led to more NonREM oscillations in the final two bins of the day. However, CBD changed the frequencies and decreased the amplitudes of these as well as quiet wake ripple oscillations.

### CBD decreases the number of long ripples

Memories are reactivated during sleep and these reactivations preferably take place during hippocampal ripples.^46^ Recently, it has been shown that extending short ripples during a task led to better working memory due to longer reactivation events.^47^ Thus, long ripples during sleep could be especially important for memory consolidation of extended experience or more complex information. Therefore, next we split detected NonREM ripples into short (≤100 ms) and long (>100 ms) events (Figure 3E). Most events were short and these presented the same time-course as all ripples, with increases after CBD in the final sleep bins (Figure 3F). In contrast, long ripples occurred less already early in the day after CBD administration (Figure 3G). CBD decreased the amplitude of both short and long ripples (Figure 3H). Interestingly, while short ripples showed significant phase coupling with slow oscillations in both VEH and CBD (both p < 0.0001), long ripples were only coupled to the slow oscillation after VEH administration and not CBD (p < 0.05, p = 0.9, Figure 3I).

Plotting the hippocampal and cortical power spectrum during short and long ripples (in comparison to random selected baseline signal) revealed a frequency independent increase in power for all events to baseline. However, in vehicles long in comparison to short ripples had an even higher power, especially in the prelimbic cortex (Figure 3J). This was not evident in CBD. The power spectrum can be used to calculate E/I balances as well as overall activity levels.^48^^,^^49^^,^^50^^,^^51^^,^^52^ Specifically, from lines fitted to the double logged (power and frequency) power spectrum the offsets and slopes can be derived (Figure 3K). The offsets reflect the overall activity (higher offset → higher activity), while the slopes reflect the E/I balance (flatter slope → higher E/I ratio). In vehicles, offsets (activity) in both brain areas increased from baseline to short and from short to long ripples, but the increase was less after CBD. Interestingly, slopes were generally higher in the hippocampus than in the cortex (higher E/I balance) and CBD increased these even more (especially in the hippocampus). After vehicle slopes decreased for ripple events (lower E/I balance). While this was similar in CBD, due to the overall higher baseline, slopes remained higher during ripples.

To replicate the finding that CBD led to shorter ripples in more animals, we performed acute experiments where animals from the behavioral experiment on their final day received either CBD or Veh, were subjected to urethane anesthesia and were implanted 32-channel silicone probes targeting the hippocampus. Ripples could be detected in the NonREM-like state and we replicated known changes^23^^,^^53^^,^^54^^,^^55^ of ripples under urethane (slower and bigger Figure S4). However, we also confirm that as in natural sleep after CBD administration ripples became shorter and smaller in comparison to vehicle (Figure 3L). Also, after splitting into short and long ripples, the same effect was seen for both types.

In sum, after CBD there were fewer long ripples and these were less locked to the slow oscillation phase. During especially long ripples, CBD showed less activity in the cortex. In general, CBD led to higher E/I ratio that could have led to the changes in ripple events.

### Only in vehicle did cumulative learning lead to more long ripples after delta waves

After establishing general effects of CBD on sleep, we next investigated the different memory training conditions in more detail. For this purpose, we analyzed NonREM oscillatory coupling, which is known to be a reliable learning marker.^28^ However, many different types of Delta-Spindle-Ripple couplings have been reported and implicated to be important for memory consolidation. These couplings can be interactions between two oscillations such as delta followed by spindle,^56^ delta followed by ripple,^57^ ripple followed by delta,^28^ and spindles with a ripple in their troughs,^58^ but also three-oscillation interactions such as delta followed by spindle with a ripple in the trough,^59^ delta followed by ripple then spindle,^60^ ripple followed by delta and then spindle.^28^ Until now, experiments tend to report only on one type of coupling, making it difficult to compare results and create a framework for potential different or common functions.

**Figure 4:**
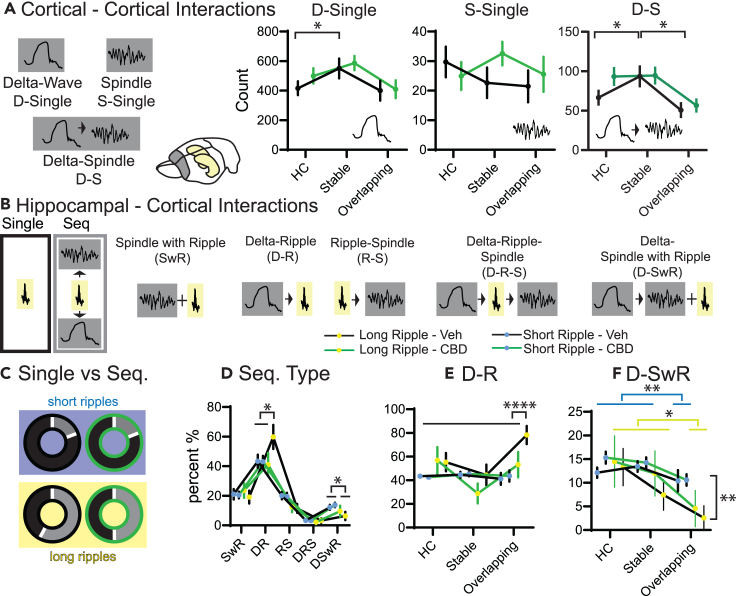
**Oscillation interactions** (A) Cortical interactions. Oscillations can be single delta (D) or spindle (S) waves, or coupled delta-spindle events (D-S). Counts of events shown for three different conditions home cage (HC), Stable and Overlapping (each rmANOVA with condition and treatment, D-single condition F_2,62_ = 3.9 p = 0.026 other p > 0.2, S-Single all p > 0.13, D-S condition F_2,62_ = 6.2 p = 0.0034, treatment F_2,62_ = 3.4 p = 0.076, interaction p = 0.29, orthogonal comparison run only for condition collapsing treatment). (B) shows different types of oscillatory coupling. (C) Fraction of short (top blue background) and long (bottom yellow background) that are part of sequences (gray) or occur alone (black). Left vehicle (black edge), right CBD (green edge). There was a significant effect (chi-square 534.2 p < 0.0001). (D) Fraction of coupled long and short ripples spread over the different interaction types (sequence types F_4,160_ = 77 p < 0.0001, sequence types X ripple types F_4,160_ = 2.3 p = 0.06, other p > 0.16). (E) As in D fraction of events that are D-R but split for the different conditions (condition F_2,204_ = 5.2 p = 0.006, ripple type F_1,204_ = 7.2 p = 0.0081, treatment F_1,204_ = 3.1 p = 0.08, condition X ripple type F_2,204_ = 7.4 p = 0.008, ripple type X treatment F_1,204_ = 3.9 p = 0.05, other p > 0.2). (F) Same for D-SwR (condition F_2,204_ = 6.5 p = 0.0018, ripple type F_1,204_ = 5.5 p = 0.019 other p > 0.18). Only in overlapping an increase was seen in vehicles but not CBD. Orthogonal comparisons ∗p < 0.05, ∗∗∗∗<0.0001 n = 4.

First, we focused on cortical events that can also be measured in human EEG experiments. Interestingly, the largest condition effect was seen for Delta-Spindle coupling (D-S), where only in the simple learning condition (Stable) there was an increase in D-S events that was not seen in single spindle events and was only weakly present for single delta events (Figure 4A).

Next, we analyzed different possible combinations of hippocampal-cortical interactions (Figure 4B): Delta-Ripple (D-R), Delta-Ripple-Spindle (D-R-S), Ripple-Spindle (R-S), Spindle with Ripple (SwR) and Delta-Spindle with Ripple (D-SwR). Overall, long ripples were more likely to occur in one of the sequences than alone. In contrast, short ripples were much less likely to occur in a sequence. After CBD, these differences remained, however, now the fraction of long ripples in sequences decreased (Figure 4C). When splitting for sequence type, it became apparent that in vehicles, but not CBD, long ripples occurred more than short ripples as D-R events. In contrast, for both CBD and vehicle short ripples occurred more as D-SwR events than long ripples (Figure 4D). Finally, since long ripples should preferentially be associated to longer reactivations and these in turn would be associated to previous knowledge, we next split the recordings for the different conditions. The increase of long ripples occurring as D-R events shown in panel D was due to a selective increase of these events after the Overlapping in comparison to the Home Cage or simple learning control (Stable) for vehicles and not CBD (Figure 4E). This condition-selective CBD effect mirrors the behavioral finding that only in Overlapping – testing complex, abstracted memory – animals showed worse performance with CBD. Interestingly, D-SwR events were less in overlapping for both short and long ripples (Figure 4F).

In sum, long ripples are more likely to occur in sequences than short ripples. After learning a complex memory (Overlapping), a selective increase in the fraction of long ripples that occurred after delta waves was seen and accompanied by a decrease in D-SwR events. The increase in D-R was absent in CBD and potentially led to the behavioral deficit in this memory condition. After the simple memory condition (Stable) an increase in D-S events was seen in both CBD and veh.

## Discussion

We investigated the effects of oral CBD intake on memory consolidation and sleep in rats. We could show that CBD extended natural sleep but also led to a higher Excitatory/Inhibitory (E/I) balance, smaller amplitude NonREM oscillations and less long ripples in NonREM sleep. We replicated the shortening of ripples after CBD in more animals recording sleep-like anesthesia states. Only after vehicle treatment, complex learning resulted in an increased fraction of long-ripples following delta waves, a potential consolidation mechanism. In CBD, this condition-specific increase in oscillation-coupling was not seen and the rats showed deficits in complex memory expression the next day. In contrast, simple learning led to an increase in delta-spindle coupling events in both vehicle and CBD, and following both treatments intact memory expression for simple memory was seen the next day. Thus, while we can confirm the beneficial effect of CBD on sleep length, this effect was modest and accompanied by changes in E/I balance and NonREM oscillations that led to a deficit in complex memory consolidation.

### CBD and E/I balance

CB1-receptors are found on the presynaptic terminals of interneurons; thus, a main neuronal effect of systemically administrated CBD should be a change in E/I ratio. Due to this, CBD is currently being handled as a novel therapeutic agent restoring E/I balance in neural models of epilepsy and autism^3^^,^^4^ in addition to being used increasingly as sleep medication.

We know that activation of the CB1-receptors with synthetic agonists inhibits hippocampal GABAergic neurotransmission and dysregulates the distribution of interneurons.^61^^,^^62^ What remains unclear is in which direction CBD administration changes the E/I ratio. Depending on the dose, CBD can have different effects as functional agonist or as antagonist of the CB1-receptor.^34^^,^^63^ Thus, actions of CBD are complex and depending on the dose, it can increase or decrease inhibition. High doses increased sleep durations^11^ and the opposite effect was seen with low doses.^12^^,^^64^

Here, we aimed to model an acute, high-dose intake, as would be recommended in humans to promote sleep.^44^ We could replicate the finding that high-dose administration increased total sleep time, even though the effect was modest and was seen as an extension of the NonREM sleep period at the end of the day. It has been previously shown that by analyzing the slopes of the power spectrum, one can determine E/I ratio.^48^^,^^49^^,^^50^^,^^51^^,^^52^^,^^65^ Interestingly, during NonREM sleep periods CBD led to an increase in E/I ratio (Figure 3K), likely by decreasing inhibition. This was visible in the prefrontal cortex but was even more evident in the hippocampus. Perhaps the change in E/I balance contributed to the extension of sleep. While a direct relationship between E/I balance and sleep is not yet known, we do know that interneuron activity is necessary for most sleep oscillations as we will expand on next.

### CBD and NonREM oscillations

NonREM sleep is dominated by the transition of up- and down-states (states of general neuronal activity and silence, respectively). Large down-states can induce a delta -wave in the LFP signal, which we can detect. Interestingly, two major classes of interneurons, the parvalbumin and the somatostatin positive cells, tightly control both up-to-down and down-to-up state transitions.^66^ The activity of these interneurons is needed to initiate and maintain the down-state.^66^ Further, sleep spindles are associated with local, cortical parvalbumin interneuron activity measured with calcium imaging,^67^ which again is potentially necessary to maintain this oscillation. Finally, hippocampal ripples are initiated by excitatory, pyramidal cell activity but then maintained by activity of interneurons.^42^ Thus, any effects of CBD on these sleep oscillations are likely due to its effect on inhibition. Already previously it had been shown that administration of CB1-agonist disrupts ripples in the hippocampus.^24^^,^^25^^,^^26^^,^^27^

Here, we could show that CBD affects ripples and other sleep oscillations as well. Deltas and NonREM ripples became slower, spindles and quiet wake ripples faster (Figures 3C and 3D). Importantly, all four oscillations became smaller in amplitude, likely due to decreased entrainment of excitatory neurons by the now attenuated interneurons. E/I ratio was changed more in the hippocampus than in the cortex during sleep and we also saw a more complex picture of CBD interactions with hippocampal NonREM ripples. During ripples normally E/I balance decreases; this decrease was seen less in CBD and, due to higher E/I baseline levels in CBD, event E/I ratio remained higher than the baseline of vehicles. A specific E/I balance is needed for ripple maintenance;^23^^,^^40^^,^^41^ correspondingly, we saw fewer long ripples in CBD. We confirmed this finding in more animals using sleep-like state recordings under anesthesia. Urethane anesthesia is a well-known model for sleep.^53^^,^^54^^,^^55^ Urethane does change sleep oscillations in comparison to their natural-sleep counterparts^23^^,^^68^ (Figure S4), however there are no known interactions with CBD.

Interestingly, those long ripples that remained in CBD showed smaller cortical, wide-band responses in the spectrogram (Figure 3J). Critically, in CBD the condition-specific increase in coupling of large down-states to long ripples (D-R sequences) after complex learning was absent (Figure 4E), likely leading to the memory deficit seen after CBD treatment in this condition. In contrast, while there were E/I differences in the cortex, these were smaller than the differences in the hippocampus. Correspondingly, there was no CBD effect in the count of cortical oscillation-coupling events (delta-spindle, Figure 4A), that were associated to simple-learning, and CBD also did not lead to deficits in this memory condition.

In sum, by changing excitatory and inhibitory neuronal balance, CBD led to changes in hippocampal-cortical oscillations and coupling resulting in deficits consolidating complex learning events. In contrast, within-cortex coupling remained intact, which likely was why CBD treated rats did not show deficits in simple learning.

### CBD and NonREM sleep

In our study, CBD led to more NonREM sleep, but this effect was modest and only visible at the end of the recording period (few hours before light off). CBD has been proposed to be a treatment for chronic pain, anxiety, epilepsy, and sleep disturbances.^1^^,^^2^^,^^4^ CBD as well as CB1-receptor agonist have been shown to promote sleep in multiple pre-clinical studies.^5^^,^^6^^,^^7^^,^^8^^,^^9^^,^^10^ A clinical study in patients with neuropsychiatric disorders showed that CBD administration improved sleep quality in more than half of the patients up to a month after administration and alleviated anxiety symptoms.^10^ However, the effect of CBD on sleep is dose-dependent with high doses increasing sleep duration^11^ and the opposite effect is observed with low doses.^12^ CBD may also have different effects in the aged population in contrast to young adults and adolescents.^13^ A recent meta-analysis criticized that rigorous, controlled evidence for the therapeutic efficacy of CBD is lacking for many health conditions, but they also found that the utility of CBD to treat epilepsy is well supported^69^ while additional controlled trials are needed to elucidate the efficacy of CBD as a sleep aid given that findings in this area appear to be mixed.^11^^,^^69^^,^^70^^,^^71^ However, in one study^11^ participants received oral placebo or CBD capsules (40, 80, or 160 mg); those receiving 160 mg CBD had a longer duration of sleep – similar to our finding – while all CBD doses decreased remembrance of dreams relative to placebo.^11^

### Oscillation-coupling for memory consolidation

At this point we do know that ripples, spindles, and delta waves in sleep are important for memory consolidation. Further, for some of their functions these oscillations are coupled to each other, as reported by many researchers. Surprisingly, no two studies seem to be in agreement, which coupling is the one to look for. Reported couplings can be interactions between two oscillations,^28^^,^^56^^,^^57^^,^^58^ but also three-oscillation interactions.^28^^,^^59^^,^^60^ Experiments tend to report only one type of oscillation, and different types of memories as well as different coupling events are never directly compared.

Here, we systematically investigated all these couplings directly comparing a non-learning control (HC) to both a simple and complex memory condition. By contrasting these different conditions, we can extrapolate, which effects are driven by which type of learning or experience.^43^^,^^72^^,^^73^ Changes induced by all OS conditions in comparison to Home Cage, should represent more general experience-dependent effects enabling simple memory consolidation or homeostasis.^43^^,^^72^^,^^73^ Changes seen only after simple learning, would represent consolidation of simple memories, already reinforced during training.^43^^,^^72^^,^^73^ In contrast, changes seen specifically after complex learning should represent semantic-like memory consolidation and the comparison and integration of new and old information.^43^^,^^72^^,^^73^

Interestingly, here we can show in vehicles that simple learning led to more Delta-Spindle events (Figure 4A). In contrast, in complex learning, the fraction of long-ripples following a delta-wave increased while the fraction of ripples occurring during a spindle after a delta wave (D-SwR) decreased (Figures 4E and 4F). We had already previously observed that the increase of delta and spindles and their coupling was linked to simple experiences,^20^ and this coupling is most often reported by researchers investigating human subjects with simple, associative memory paradigms such as paired-associative word-list or reinforced spatial learning.^59^^,^^74^ In contrast, ripples following delta are reported, for example, in experiments focused on prelimbic reactivations when animals learn complex rules.^57^ It would be tempting to speculate that Delta-Spindle and Spindle with Ripple Coupling would facilitate simple learning while Delta-Ripple would correspond to consolidation of complex memories relying during consolidation on the comparison to other previously encoded experiences. Our current results support this, since after CBD the condition-specific Delta-Ripple increase was absent but Delta-Spindle coupling remained the same, and correspondingly CBD only induced a memory deficit in the complex and not simple memory condition.

**Figure 5:**
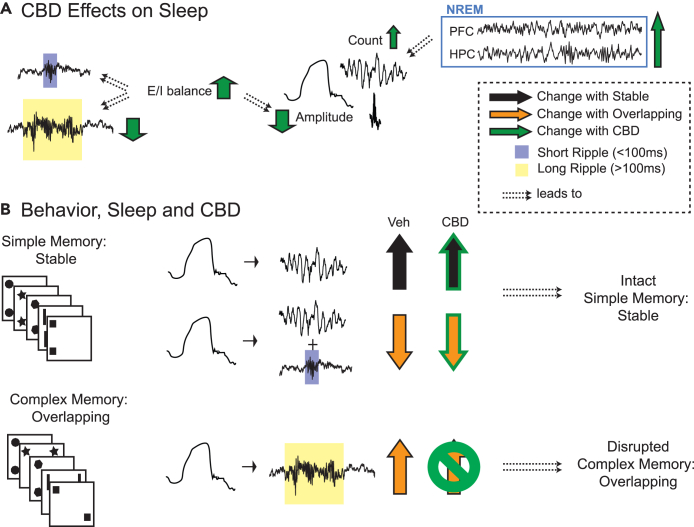
**Summary** (A) General CBD effects on sleep. CBD increases excitatory/inhibitory balance (E/I), which (potentially) leads to less long ripples and smaller NonREM oscillations. CBD also extends NonREM sleep and thus increases the number of NonREM oscillations at the end of the sleep period. (B) Behavior, Sleep and CBD effects. After simple memory training (condition Stable) there is an increase in Delta-Spindle events in vehicle (VEH), which is unchanged in CBD. After complex memory training (condition Overlapping) there is a higher fraction of long-ripples following delta waves, but a smaller fraction of both short and long ripples during spindles following delta waves. The former but not latter effect is abolished with CBD, leading to disrupted complex memory consolidation in CBD.

In conclusion, by combining oral CBD administration with the object space task and electrophysiological recordings, we could show that CBD leads to (1) extension of NonREM sleep, (2) increased E/I balance, (3) smaller NonREM oscillations, and (4) fewer long ripples and less delta-long ripple coupling (Figure 5). The latter leads to (5) deficits in complexmemory consolidation but simple memory remains intact after CBD in young adult rats.

### Limitations of the study

There are some limitations to be considered in this study. While most ripples occurred during sleep, some were during quiet rest and these also showed changes with CBD. Thus, we cannot conclusively determine if the effect of CBD on memory consolidation is solely due to sleep or also consolidation during rest. Here, we examine the role of an acute high dose CBD known to be beneficial for sleep. However, many users would use a chronic dosage which may show different interactions with sleep and memory.

We confirmed our ripple findings with recordings under anesthesia. While urethane anesthesia is a known model for sleep-like stages and oscillations, there are known differences that we also see here (Figure S4). However, there was no interaction with CBD and we did replicate the findings shown in natural sleep recordings.

Finally, we investigated young adult rats (3–4 months at start of experiment). Due to potential differential effects of age of CBD,^9^^,^^13^ these results may be specific to our targeted age group.

## STAR★Methods

### Key resources table

**Table:** 

REAGENT or RESOURCE	SOURCE	IDENTIFIER
**Chemicals, peptides, and recombinant proteins**		

CBD	CBD depot	https://www.cbdepot.eu/products/cannabidiolum-gmp
Urethane	Sigma Aldrich	https://www.sigmaaldrich.com/ZA/en/product/aldrich/94300
Cresyl violet acetate	Sigma Aldrich	https://www.sigmaaldrich.com/ZA/en/product/sigma/c5042
DPX mounting medium	VWR	https://nl.vwr.com/store/product/9677593/non-aqueous-dpx-mounting-medium

**Deposited data**		

CBD chronic recordings LFP data	This paper	https://osf.io./fwshb/
CBD acute recordings LFP data	This paper	https://osf.io/htmqu/

**Experimental models: Organisms/strains**		

Lister-hooded rats	Charles Spring River Laboratories	

**Software and algorithms**		

CBD scripts repository	This paper	https://github.com/genzellab/cbd
MATLAB	The MathWorks Inc.^75^	https://www.mathworks.com
Freely Moving Animal Toolbox	Zugaro^76^	https://fmatoolbox.sourceforge.net/
Fieldtrip	Oostenveld et al.^77^	http://fieldtriptoolbox.org
Sleep architecture analysis	Aleman-Zapata & Navarro-Lobato^78^	https://github.com/genzellab/sleep_architecture
TheStateEditor	Grosmark^79^	https://github.com/buzsakilab/buzcode/ https://doi.org/10.7282/T3K072J1
SPSS	IBM Corp.^80^	https://www.ibm.com/products/spss-statistics
FOOOF toolbox	Donoghue et al.^65^	https://fooof-tools.github.io/fooof/

### Resource availability

#### Lead contact

Further information and requests for resources and reagents should be directed to and will be fulfilled by the lead contact, Lisa Genzel (Lisa.Genzel@donders.ru.nl).

#### Materials availability

This study did not generate new unique reagents.

#### Data and code availability

• A downsampled version of the chronic and acute electrophysiological data has been deposited at Open Science Framework (OSF) and is publicly available as of the date of publication. The DOIs are listed in the key resources table.
• All original code has been deposited at Github and is publicly available as of the date of publication. The DOI is listed in the key resources table.
• Any additional information required to reanalyze the data reported in this paper is available from the lead contact upon request.

### Experimental model and study participant details

#### Animals

Six-to eight-week-old male Lister Hooded rats weighing between 250 and 300 g at the start of the experiment (Charles Rivers, Germany) were used in this study. They were housed in conventional Eurostandard type IV cages (Techniplastic, UK) in a temperature-controlled (20 + 2°C) room following a 12 h light/dark cycle with water and food provided *ad libitum*. A total of 48 rats were used in this experiment: 44 in the behavioral experiment (3 sub batches of n = 16, 14 and 14), and four for chronic electrophysiological recordings (two more served as implant-pilots but were not included in the study). The behavioral experiments and electrophysiological recordings were performed during the light period (between 9:00-18:00). All animal procedures were approved by the Central Commissie Dierproeven (CCD) and conducted according to the Experiments on Animals Act (protocol codes, 2016-014-024 and 2020-0020-006).

### Method details

#### Study design

A total of 48 rats were used in this study: 44 rats were used for behavioral experiments and four rats were used for chronic electrophysiological recordings. All rats were first extensively handled for multiple days until they experienced minimal stress while working with the experimenters (see handling videos on www.genzellab.com). The first batch of 44 rats was first habituated to the oral feeding regime and the behavioral training box for a week. In the following week, they were orally administered with >98% *trans*-cannabidiol (CBD) or vehicle (VEH) (counterbalanced across animals) and trained in the Stable and Overlapping condition of the Object Space task (all conditions run cross-over within each animal) in smaller sub batches of 14–16 animals (maximum 8 in one day).

The 4 implanted rats underwent surgery for wire drive implantation. After surgery, they were allowed to recover for up to 2–3 weeks, during which the wire arrays were slowly lowered to reach the pyramidal layer in the CA1 region of the hippocampus and the rats were habituated to the behavior training box and the oral feeding regime. Upon reaching the target, we started the behavioral experiments. Similar to the behavior batch, these rats were orally administered with either CBD or VEH and then trained in the Object Space Task (all conditions cross-over within subject). Brain activity was recorded during exploration and rest periods.

#### Animals

Six-to eight-week-old male Lister Hooded rats weighing between 250 and 300 g at the start of the experiment (Charles Rivers, Germany) were used in this study. They were pair-housed in conventional Eurostandard type IV cages (Techniplastic, UK) in a temperature-controlled (20 + 2°C) room following a 12 h light/dark cycle with water and food provided *ad libitum*. The first batch of 44 rats was kept under these conditions until the end of the experiment. The batch of chronic rats were single-housed after wire drive implantation and kept in these conditions until the end of the experiment. A total of 48 rats were used in this experiment: 44 in the behavioral experiment (3 sub batches of n = 16, 14 and 14), and four for chronic electrophysiological recordings (two more served as implant-pilots but were not included in the study). The behavioral experiments and electrophysiological recordings were performed during the light period (between 9:00-18:00). All animal procedures were approved by the Central Commissie Dierproeven (CCD) and conducted according to the Experiments on Animals Act (protocol codes, 2016-014-024 and 2020-0020-006).

#### CBD

(−)-trans-Cannabidiol (CBD, >98%) was obtained (https://www.cbdepot.eu/products/cannabidiolum-gmp) for all experiments. Rats were treated with either CBD (120 mg/kg in 300 μL flavored olive oil, p.o.) or vehicle (300 μL flavored olive oil, p.o.). Different flavoring agents, namely, vanilla, cinnamon, star anise and clove, were used to make multiple flavors of olive oil, which were then used to mask the taste of CBD, so that the rats would always be naive to what they were fed. The use of all flavors was counterbalanced, such that each rat received all flavors for both CBD and VEH. CBD solution was always freshly prepared in one of the flavored oils prior to oral administration. In order to prepare the solution, first the amount of CBD to be administered was determined based on the rat’s weight and the compound was weighed accordingly. The flavored oil was heated up to a temperature of 50°C–60°C and then the CBD compound was slowly dissolved into it. This process would take a few minutes until a clear solution was obtained, which is indistinguishable from vehicle oil. Individual syringes with 300 μL CBD or VEH were prepared for each rat. The experimenter who performed the oral administration was blinded to the treatment each rat received, and the drug was always administered an hour before the start of behavioral training. Further, the experimenters performing the behavioral training and electrophysiological recordings were blinded to the treatment of the animal as well. Previous studies have shown oral administration of CBD to be effective in crossing the blood brain barrier and plasma concentrations are shown to reach their maximum around 4–6 h after oral administration, which is optimum time window to examine its effect on memory consolidation.^44^^,^^45^

#### Behavior - object space task

The Object Space Task was used in this study to assess the effects of CBD on simple and semantic-like memories.^43^ This task is based on the tendency of rats to explore novel object locations in an open field arena across multiple trials. The animals were extensively handled by the experimenters in the first week to minimize their stress levels during the interactions. Next, they were habituated to the training box (75 × 75 cm) for five sessions over five days. On the first day, they spent 30 min in the training box with their cage mates. On the second and third days, each rat individually spent 10 min exploring the training box. For the final 2 days, two objects (made from DUPLO blocks, not used in main experiment) were placed in the center of the box and the rats were allowed to explore for 10 min each day.

A total of 44 rats were trained in the Stable and Overlapping conditions of the task and ran in smaller sub batches of 8. The condition sequence and object locations were counterbalanced across all rats and the experimenter was blinded to the conditions and drug treatment. We followed a within-subject design, wherein every rat performed all conditions of the task with CBD and VEH.

For rats, running one round of one condition took two days – a training day and a test day 24 h later. The rats were first orally administered CBD or VEH and then started with behavioral training an hour after drug administration. The training day consisted of five trials, 5 min each with an inter-trial interval of ∼45–50 min. During each trial, the rat was placed in the training box to explore a pair of identical objects at fixed locations. The object pairs were changed from trial to trial. Additionally, to prepare for one session of training and test, the training box was equipped with 2D and 3D cues to create a unique spatial environment for the rats to orient themselves. This box setup remained the same for one session of training and test, and was changed as much as possible for the next session, in order to create a distinctly different environment each time. In the Stable condition, the positions of the objects remained the same across all trials. 24 h later, during the test trial, one object was moved to a different position. This condition allowed us to assess simple memory in the rats. For training in the Overlapping condition, one position of the object remained constant across all trials whereas the other kept changing. However, the object positions in the final trial of the training day and the test day, 24 h later, were kept the same. If the rat formed a cumulative memory of one object position constantly moving across multiple trials on the training day, he would spend more time exploring that position. This would let us assess semantic like memory in the rats. Implanted animals additionally had Home Cage days, where they would have the same daily regime as on a training day but instead of being placed in the training box for the 5 min training periods, the animal was kept awake in the home cage. Therefore, these days are non-learning controls that are controlled for general wake activity/sleep disturbances of training and time of day.

All behavior sessions were recorded using a webcam placed above the training box. The object exploration times were scored online in real time during the trials using a scorer program developed in the lab for training and scoring (https://github.com/NeuroNetMem/score). Whenever a rat would sniff or climb on or interact with an object, it was scored as an exploring behavior. Obsessive chewing or biting of an object was not considered as an exploratory behavior, and extra care was taken to avoid using objects that would trigger these behaviors. The scoring data from the behavior sessions was saved in an Excel sheet and was used for further analyses.

#### Bilateral wire drive implants

A customized lightweight wire drive implant was manufactured in collaboration with 3Dneuro (https://www.3dneuro.com/) to implant bilateral wire arrays in the prelimbic cortex and the CA1 region of the hippocampus. Customized individual wire arrays (Science Products, catalog no. NC7620F) were first built for each brain region. The wire arrays for the prelimbic cortex consisted of four wires of the same length glued to each other; for the hippocampus, the array consisted of four wires of different lengths with a 70° angle between them to enable recording from the pyramidal layer as well as from below and above the layer. These arrays were then glued to polyamide tubes which were glued to the 3D printed drive body. The drive body was designed according to the Rat Brain Atlas in Stereotaxic Coordinates^81^ to ensure the correct placement of arrays in the regions of interest. Additionally, a shuttle and screw were included in the drive body design for the hippocampal arrays, so that they could be turned down after implantation to reach the target layer. Finally, the drive body was designed to enable recording of each region from both hemispheres. Once all the arrays were loaded into the drive body, their free ends were connected to a customized 32 channel electrode interface board (EIB) using gold pins (Neuralynx). A single NPD dual-row 32 contact omnetics connector was attached to the EIB to connect the headstage for later recordings. The hippocampal wire arrays were flushed with the polyamide tubes before implantation. The bottom of the drive was deepened in 70% ethanol for at least 2 h before implantion into the brain.

#### Drive implant surgery

Shortly before the start of surgery, all rats received a subcutaneous (sc) injection of carprofen (5 mg/kg) to serve as an analgesic. After placing the rats into the stereotaxis, they received a subcutaneous injection of a mixture of 4 mg/kg lidocaine and 1 mg/kg bupivacaine in a 0.9% NaCl physiological serum locally at the skin surface above the skull as a local analgesic. Finally, they also received a 2 mL of 0.9% NaCl physiological serum subcutaneous injection at the start and end of surgery. Since this was a recovery surgery, utmost care was taken to maintain the most sterile conditions possible and the entire surgery was performed under isoflurane inhalation anesthesia. Additionally, the rats were administered a 10 mg/kg subcutaneous injection of Baytril antibiotic at the beginning of surgery to prevent postsurgical infections. Two pairs of holes were drilled bilaterally to reach the following targets – prelimbic cortex (AP +3.5 mm and ML + -0.5 mm) and hippocampus (AP -3.8 mm and ML + - 2 mm). Additionally, a ground screw (M1x3 mm) was placed on the right hemisphere of the cerebellum. Three more M1x3 mm supporting screws were placed and bound to the skull using Superbond C&B dental cement (Sun Medical, Japan). Upon drilling all holes above the target regions, the dura mater was carefully removed, exposing the surface of the brain. Finally, the wire-drive was carefully positioned on the brain’s surface, such that the wire arrays fit well in the drilled holes. On finalizing the position, the drive was attached to the skull and screws by simplex rapid dental cement (Kemdent, UK). The wire arrays for HPC were slowly lowered (∼1.5 mm DV from brain surface) to a layer close to the pyramidal layer in the CA1 region. The pyramidal layer was reached progressively in the subsequent days during signal checks in the rats’ recovery period. With one out of the 4 rats, we were not able to reach the pyramidal layer. This rat was thus included only for analysis of cortical events and sleep architecture analyses.

#### *In vivo* electrophysiological recordings

The animals were closely monitored for a week following the drive implant surgery to ensure they had a good recovery. Their weights were monitored daily during this period to ensure a stable growth curve. The rats were allowed to recover for a couple of weeks following surgery before starting with recordings. Prior to the surgery, the rats were habituated to the sleep boxes, chocolate treats and the oral feeding regime, to minimize any stress post-surgery. During the recovery period, they were re-introduced to the sleeping boxes and their local field potential (LFP) activity was monitored during wake and rest periods to assess the placement of the wire arrays. The HPC arrays were further lowered in small steps during this period and the correct placement was confirmed with the LFP activity. Finally, after a week of recovery, the rats were also habituated to the Object Space box according to the habituation protocol described above.

Once all arrays were in the target regions, recordings were started while the rats were being trained in the Object Space task and during the in between rest periods. The feeding and training protocol that was followed here was similar to the one described above. Each rat had a session per experimental condition (Homecage, Stable, Overlapping) with prior administration of CBD or VEH. In the end, every rat ran each session, once with CBD and another with VEH. The sequence of conditions was counterbalanced for every rat and the experimenter scoring the behavior was blinded to which drug treatment the rat was assigned to.

The LFP and accelerometer activities detected by the channels were amplified, filtered and digitized through a 32 channel headstage (IntanTechnology) connected through an Intan cable and a commutator into the Open Ephys acquisition box.^82^ The signal was visualized using the open source Open Ephys GUI at a sampling rate of 1 Hz–30 kHz.

##### Electrolytic lesions

After the final recording session, the animals received electrolytic lesions under isoflurane inhalation anesthesia 48 h before perfusion to identify the electrode tips placement. To this end, a current of 8 μA for 10 s was applied in two wires per array using a stimulator.

#### Acute recordings

At the end of behavior recordings, 30 rats were used to carry out acute electrophysiological recordings to monitor the effects of CBD on sleep like states under anesthesia using silicon probes. The rats would receive an oral administration of CBD or VEH (120 mg/kg) at ∼10 a.m. and 30 min later would receive an i.p. injection of urethane anesthesia (1.4 g/kg). After injection, we would wait for the next 30 min for the anesthesia to set in and start with surgery at ∼11 a.m. The aim was to start with surgery an hour after oral administration of CBD/VEH.

#### Stererotaxic surgery

Shortly before the start of surgery, all rats received an s.c injection of carprofen (5 mg/kg) to serve as an analgesic. On setting the rat into the stererotax, they further received an s.c. injection of a mix of 4 mg/kg lidocaine and 1 mg/kg bupivacaine in a 0.9% NaCl physiological serum locally at the skin surface above the skull as a local analgesic. Lastly they also received a 2 mL of 0.9% NaCl physiological serum s.c. injection at the start and end of surgery. The target areas for the recordings were medial prefrontal cortex (mPFC) and hippocampus (HPC) with the following coordinates – AP = 3.5 mm, ML = 0.5 mm and DV = 2.6 mm (from brain surface) for PFC and AP = −3.2 mm, ML = 2 mm and DV = 4.3 mm (from brain surface) for HPC. All coordinates were calculated with respect to standard bregma and lambda coordinates (BÜTTNER-ENNEVER, 1997). Two craniotomies (2 × 1 mm and 1 × 1 mm for PFC and HPC, respectively) were drilled above the target areas on the right hemisphere and a hole in the left cerebellum for the ground screw. Finally, once the craniotomies were cleaned and the dura mater was clearly visible, the silicon probes were placed in position and lowered slowly to both targets. The tips of the probes were coated with DIL stain (catalog no. D282) before lowering to facilitate better visualization of the electrode damage in the target region during histology.

#### Recordings

The surgery setup was enclosed in a Faraday cage to prevent any electrical noise in the recordings. Both silicon probes (32 channels in each probe) were connected to an OpenEphys acquisition box (Siegle et al., 2017). The signal was visualized using the OpenEphys GUI and recorded at a sampling rate of 30 kHz. Signals from both brain regions were recorded for 6–7 h and we checked every hour whether the craniotomies were hydrated and the temperature of the heatpad remained constant. At the end of the recordings, the animals were sacrificed via transcardial perfusion and the brains were extracted for histology analyses.

#### Histology

##### Brain processing

Rats were sacrificed via transcardial perfusion 48 h after the electrolytic lesions. They were overdosed with 150 mg/kg sodium pentobarbitol ip prior to perfusion. They were perfused first with 100 mL of 0.1 M phosphate-buffered saline pH 7.4 (PBS) followed by 250 mL of 4% paraformaldehyde (PFA) made in 0.1 M PBS. After extracting the brains, they were kept overnight in PFA at 4°C. The brains were rinsed in 0.1 M PBS the next day (3 × 10 min) and then kept in a solution of 30% sucrose, 0.02% NaN_3_ in PBS for cryoprotection. Once the brains sank to the bottom of the vial, they were frozen in dry ice and stored for the long term in a −80°C freezer. For further processing, the brains were sectioned in a cryostat (SLEE medical, Germany) and 50 micron coronal sections of target regions were obtained and collected in 48-well plates containing 0.02% NaN_3_ in PBS and stored at 4°C.

##### Nissl staining

Coronal sections containing the prelimbic cortex (PFC) and hippocampus (HPC) were sequentially mounted (in increasing AP coordinates) on gelatin-coated slides and incubated overnight at 37°C. The following day, the slides were further processed for Nissl staining. Slides were first hydrated in 0.1 M PBS and then in Milli Q water for 20 min each. Next, the slides were stained in 0.7% acetate Cresyl Violet for 20 min and dehydrated in an increasing ethanol gradient (water for 3 min, 70% ethanol for 20 s, 96% ethanol + acetic acid for 45 s, 100% ethanol for 5 min). Finally, in the last step, the slides were immersed in xylene for 15 min and then then mounted with DePeX mounting medium and covered with coverslip. Lesions from wire arrays were then observed under a bright field microscope (LEICA DM IRE2) and images were taken in 5X and 10X magnification.

#### Behavioral data analyses

The amount of time the rats spent exploring objects was scored in real time which would then get saved in an excel sheet. The total exploration time was calculated as the sum of time spent exploring both objects. Further on the discrimination index (DI) was calculated by subtracting the amount of time exploring the familiar location from the novel location and dividing by the total exploration time. DI > 0 indicated a preference for the novel object location, which was used as a prime measure for memory performance. DI = 0 indicated no preference for either location and finally DI < 0 indicated a preference for the stable object location. Data was analyzed with rmANOVA (within subject factors treatment, condition).

#### Local field potential analysis

We hereby describe the methods followed to process and analyze the local field potential data acquired during chronic and acute recordings.

##### Scoring of sleep in chronic recordings

Chronic recordings from the rest periods across all study days were further classified into different sleep/wake states using a sleep scoring GUI called TheStateEditor in MATLAB developed by Dr. Andres Grosmark.^79^ As the first step, one PFC and HPC channel and three accelerometer channels were selected per animal as input into the scorer interface. One could then visualize the frequency spectrograms and bandpass filtered LFP signal per brain region and the motion spectrogram during the recording period. Using this information, an experienced researcher scored the recording data in increments of 1 s epochs into one of the following states – wakefulness; NonREM; Intermediate; REM. The researcher scoring the datasets was blinded to condition and treatment for that respective study day. Absence of movements in certain periods in the motion spectrogram further aided in discriminating between sleep and wake periods. Desynchronized activity in PFC and HPC accompanied with movement in the motion spectrogram was classified as a wake state. NonREM sleep was classified when slow oscillations (0.5–4 Hz) were detected in the PFC channel accompanied by lack of movement in the motion spectrogram. REM sleep was classified when we detected desynchronized activity in the PFC channel and dominant periodic theta activity (6–8 Hz) in the HPC channel accompanied by lack of movement in the motion spectrogram. Intermediate sleep was defined as short transition periods between NonREM and REM states which were characterized by irregular theta activity in the HPC channel and high frequency desynchronized activity in the PFC channel accompanied by lack of movement in the motion spectrogram.

##### Scoring of sleep-like states in acute recordings

Sleep-like stages were classified from the recordings of anesthetized rats. Considering the absence of wakefulness during anesthesia, an automatic state classifier was designed to detect NonREM-like and REM-like states based on the spectral features of the cortical and hippocampal recordings. The spectral power of selected hippocampus and prefrontal cortex channels was estimated from 0 to 100 Hz, with a 0.5 Hz step, using a multitaper filter with a time-bandwidth product of 4 in 10-second-long epochs. Epochs with artifacts were detected using the isoutlier MATLAB function on the absolute value of the epochs’ amplitudes. Artifacts were removed and blanked with the mean of the signal. A Principal component analysis (PCA) was employed to identify spectral features of the epochs that characterized the two sleep-like states and explained most of the variance in the data. The following parameters were used as an input for the PCA: Slow oscillation power (0.1–1 Hz), delta power (1–3 Hz), theta power (3–6 Hz), low beta power (10–20 Hz), low gamma power (30–45 Hz), high gamma power (55–80 Hz), ripple power (90–300 Hz), theta-slow oscillation ratio and amplitude of the 10-second-long epoch. The frequency ranges used here and in the ripple detection were intentionally lower than those reported in the literature since urethane anesthesia has been shown to slow down the brain activity (Murillo-Rodriguez et al., 1998). The first and second principal components were kept, given that they explained most of the variance, and a K-means clustering algorithm with two partitions was computed in the PC1-PC2 state space to identify the epochs that belonged to the NonREM-like and the REM-like state clusters. The first PCA component (PC1) contained high weights for features like the epoch amplitude and slow oscillation power in both brain areas, which are expected to be high in the NonREM stage during natural sleep. Therefore, the cluster of epochs found containing higher values for PC1 was labeled as NonREM-like sleep, while the remaining cluster was labeled as REM-like sleep.

##### Sleep architecture analysis

Based on the manual scoring of the states, we computed the total sleep time (TST), wake, and total time spent in different sleep states (NonREM, REM and Intermediate) per session in MATLAB. Averages for these variables were further calculated across all sessions per rat and the mean and SEM was computed per treatment. Additionally, % of TST spent in NonREM, REM and intermediate was calculated in 45 min time bins for the 3-h rest periods after the final trial. Furthermore, the distribution of bout duration per sleep stage was calculated for every session across different treatment groups. A bout was defined as a continuous period spent in a specific sleep state and only bouts longer than 4 s were considered for analysis. The state of switching from one sleep stage to another was defined as transitions. Using MATLAB, we computed the number of bouts per sleep stage, the duration of the bouts, and transitions between sleep stages, for example, number of NonREM-REM and REM-NonREM transitions. This was computed per session for each rat and later averaged for CBD and VEH treatment groups across rats. The MATLAB scripts for this analysis can be found at https://github.com/genzellab/sleep_architecture.^78^

##### Preprocessing of chronic recordings

A prelimbic cortex channel with large slow oscillations during NonREM sleep was selected per rat. A hippocampal channel from an electrode placed in the pyramidal layer was selected based on the quality of ripples visually detected on the channel. The channels were downsampled from 30 kHz to 2.5 kHz by first employing a 3rd order zero-phase Butterworth low pass filter with a cutoff of 1.25 kHz to prevent aliasing of the signal. Artifacts observed during NonREM bouts were subject to a blanking method, which consisted of applying an amplitude threshold determined visually. Artifacts crossing the threshold were “blanked” by replacing them with the mean value of the NonREM bout. For some types of artifacts, a preceding build-up period of 1 s was also blanked. The artifact removal was done after the signal had already been bandpass-filtered in the corresponding frequency range used for the detection of ripples, spindles or delta events. This prevented artifacts due to discontinuities.

##### Preprocessing of acute recordings

The best channel in the hippocampal CA1 pyramidal layer was chosen per rat. The selection criteria consisted of selecting the channels in which ripples were visually more prominent. The channels were downsampled from 30 kHz to 600 Hz to be used for local field potential analysis. A 3rd order Butterworth low pass zero-phase filter with a cutoff of 300 Hz was used to prevent aliasing before downsampling the signal. The first 15 min of the recordings were discarded to control the brain signal instability after the probe implantation. Artifacts were detected and removed by applying thresholds to the sum of absolute values of the unfiltered recordings for the selected hippocampal and prefrontal channels. When an artifact was detected, a buildup of 0.5 s prior to the artifact and a washout period of 3.5 s after the artifact were removed and replaced with the mean value of the artifact-free signal. Before “blanking” the artifact, we bandpass-filtered the channels which would be used for the detection of ripples in the 90–200 Hz range. This was made to prevent the addition of spurious high frequency events which occur due to filtering signal discontinuities after the artifact blanking.

##### Detection of hippocampal ripples in chronic recordings

To detect NonREM ripples the downsampled channels (2.5 kHz) of the hippocampal pyramidal layer were loaded into the MATLAB workspace and their NonREM bouts were extracted. Using a 3^rd^ order zero-phase Butterworth bandpass filter, the NonREM epochs of HPC signal were filtered to a frequency range of 100–300 Hz. A custom MATLAB function was used for detecting the start, peak and end of the ripples by thresholding voltage peaks which lasted a minimum duration of 20 ms above the threshold. The start and end of the ripple were determined as half the value of the detection threshold. A closeness threshold of 20 ms was used to count ripples occurring within the proximity of each other as a single event. The detection threshold was determined by computing the standard deviations of concatenated NonREM bouts individually for pre and post sleep trials in a study day. These standard deviations were multiplied by a factor of 5 and an average was calculated to find a single detection threshold per study day. An offset of 5 μV was added to the threshold to reduce false positives. Only pre- and post-trial sleep periods with more than 3 min of NonREM were included. This process was repeated for all study days pertaining to all rats in both treatment groups. The detections were then grouped as long (>100 ms) or short (≤100 ms) ripples based on their duration, in accordance with Fernandez-Ruiz et al. 2019.To detect quiet wake ripples a similar procedure was followed, with the exception that we used signal bouts from the pre- and post-sleep periods manually labeled as wakefulness instead of NonREM.Moreover, we included thresholds for the resultant accelerometer signals, theta power (5–10 Hz) and high-amplitude artifacts to remove epochs that could lead to false positive detections due to movement or noise.

##### Detection of hippocampal ripples in acute recordings

To detect hippocampal events, we used the bandpassed channels of hippocampus CA1 pyramidal layer (90–200 Hz). Both raw and filtered versions of the hippocampal channels were displayed in a graphical user interface, which displayed an amplitude threshold for ripples on the filtered CA1 pyramidal layer channel. Thresholds were adjusted for each rat after visually verifying the correct detection of ripples. The threshold values were set in terms of the floating-point number of standard deviations with respect to the mean of the filtered signals. All selected thresholds were close to five times the standard deviation of the filtered hippocampal signal. Once the thresholds were determined, we ran the detection of ripples by thresholding voltage peaks which lasted a minimum duration of 50 ms above the threshold. The start and end of the ripple were determined as half the value of the detection threshold. A closeness threshold of 80 ms was used to count ripples occurring within the proximity of each other as a single event. We only used the detections that occurred during the NonREM-like stage.

##### Detection of cortical spindles and delta waves

A downsampled prelimbic cortex channel (2.5 kHz) with large slow oscillations was selected and using a 3^rd^ order zero-phase Butterworth filter the signal was filtered to 9–20 Hz for detecting spindles and to 1-6 Hz for detecting delta waves. The NonREM bouts were then extracted from the filtered signal and concatenated. The functions FindSpindles and FindDeltaWaves from the Freely Moving Animal (FMA) toolbox (http://fmatoolbox.sourceforge.net)^76^ were modified to adapt the thresholds for optimal detections and were then used to detect the start, peak and end of spindles and delta waves, respectively. The optimal threshold was found for each rat by visually inspecting the detections and modifying the default parameters of the functions when needed. The results were saved as timestamps in seconds with respect to the concatenated NonREM signal. They were then used to find the timestamps with respect to the recorded signal. This process was repeated for pre- and post-trial sleep periods with more than 3 min of NonREM in study days pertaining to all rats in both treatment groups.

##### Detection of oscillation sequences

The sequences between ripples, spindles and delta waves were counted in various combinations to study cortico-hippocampal coupling during NonREM sleep as done by^28^ Maingret et al. 2016. The time difference between the peaks of these events was compared to a fixed duration to establish if there was a sequential relationship in the following combinations of oscillations: Delta-Spindle (D-S), Delta-Ripple (D-R), Ripple-Spindle (R-S), Delta-Ripple-Spindle (D-R-S), Delta-Spindle co-occurring with Ripple (D-SwR). For D-S a sequence was considered when the interevent interval was between 100 and 1300 ms, for D-R it was 50–400 ms and for R-S it was 2–1000 ms. To find D-R-S sequences, the results of D-R and R-S were compared to find ripples preceded by a delta wave and followed by a spindle. To find the D-SwR sequences, the results of D-S and spindles co-occurring with ripples (see next subsection) were matched to find spindles preceded by a delta and co-occurring with a ripple. The results were saved as counts of each sequence for each pre- and post-sleep trial. This analysis was then repeated using timestamps of long and short ripples separately.

##### Co-occurrence between ripples and spindles

The co-occurrence between ripples and spindles was computed by comparing the start and end timestamps of both events. To consider co-occurrence between a ripple and a spindle, either one of the following conditions had to be fulfilled: 1) A ripple had to start and end within the duration of the spindle. 2) One of the events had to start or end within the duration of the other. Given that more than one ripple could co-occur with the same spindle, we counted separately ripples co-occurring with spindles and spindles co-occurring with ripples. This analysis was then repeated using timestamps of long and short ripples separately.

##### Slow oscillation phase analysis

The downsampled PFC signal (2.5 kHz) was filtered to the 0.5–4 Hz range using a 3^rd^ order Butterworth zero-phase bandpass filter and its Hilbert transform was computed to find the phase angle of slow wave oscillations in the range of 0°–360°. The peaks of long and short ripples were then used to find the corresponding phase and this process was repeated for all study days pertaining to all rats.

##### Oscillations characteristics

The traces of each detected event (ripples, spindles, delta waves) were extracted using the start and end timestamps obtained from the detectors. The traces of the events were filtered in their corresponding detection frequency band. Characteristics such as the amplitude and mean frequency were calculated for these filtered events using built-in and custom MATLAB functions. Namely, the amplitude of the events was calculated by computing the envelope of the filtered trace using a Hilbert transform. The absolute value of the result was taken and its maximum was found. The mean frequency of the filtered traces was computed using the meanfreq function of MATLAB.

##### Power spectra & power spectral density slope and offset analysis

Power spectra of HPC and PFC and slope/offset analysis of power spectral density (PSD) were computed for chronic recordings. They were computed for both VEH and CBD treatments. Baselines were randomly selected NonREM periods of 4 s. First, the Fieldtrip toolbox^77^ was used to compute PSD. PSD was computed from 0 to 100 Hz in steps of 0.25 Hz. A Hanning window taper was used with a length of 4 s and a 0.25-s overlap. Line noise of 50 Hz was removed using a Notch filter. To generate power spectra, the PSD values were computed as logarithms with a base of 10. To calculate the slope and offset, the ‘Fitting Oscillations and One-Over-f (FOOOF)’ Python toolbox was used (Donoghue et al., 2020).^65^ The minimum peak height threshold was set to 0.05 (units of power – same as the input spectrum), and peak width limits were between 1 Hz and 8 Hz. The outputs were in logarithms with a base of 10.

### Quantification and statistical analysis

Statistical tests reported with the results, parametric (ANOVA) and non-parametric analysis were used accordingly. The resulting values of spectral slopes and offsets were analyzed using IBM SPSS Statistics (Version 29).^80^ For slopes and offsets, a two-way ANOVA was conducted to determine the effects of treatment (VEH & CBD) and the ripple type (random NonREM baseline, short & long ripples). Statistical significance was accepted at the p < 0.05 level for simple interactions and simple main effects. Post hoc comparisons using the Tukey HSD test were conducted to investigate interaction effects.
