# Supplementary material for: CBD lengthens sleep but shortens ripples and leads to intact simple but worse cumulative memory
Source: iScience. 2023 Oct 24;26(11):108327. doi: 10.1016/j.isci.2023.108327 (PMC10656268; doi:10.1016/j.isci.2023.108327)
Supplement: Document S1. Figures S1–S4 [file mmc1.pdf]

iScience, Volume *26*

Supplemental information

CBD lengthens sleep but shortens ripples and leads to intact simple but worse cumulative memory

Anumita Samanta, Adrian Aleman-Zapata, Kopal Agarwal, Pelin Özsezer, Alejandra Alonso, Jacqueline van der Meij, Abdelrahman Rayan, Irene Navarro-Lobato, and Lisa Genzel

CBD lengthens sleep but shortens ripples and leads to intact simple but worse cumulative memory – Supplemental Materials

Anumita Samanta, Adrian Aleman-Zapata, Kopal Agarwal, Pelin Özsezer, Alejandra Alonso, Jacqueline van der Meij, Abdelrahman Rayan, Irene Navarro-Lobato, Lisa Genzel

Supplement Figures

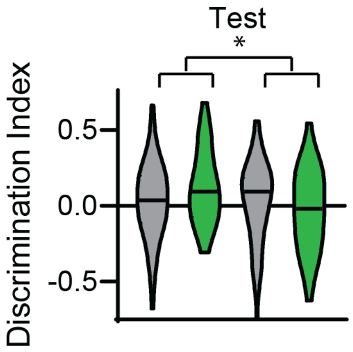

Fig. S1: Shown is the test data for all rats, related to Fig. 1.
The interaction CBD and Condition remained marginal significant (condition $F_{1,43}$=5.0 p=0.03 treatment $F_{1,43}$=0.22 p=0.6 interaction $F_{1,43}$=3.0 p=0.089).

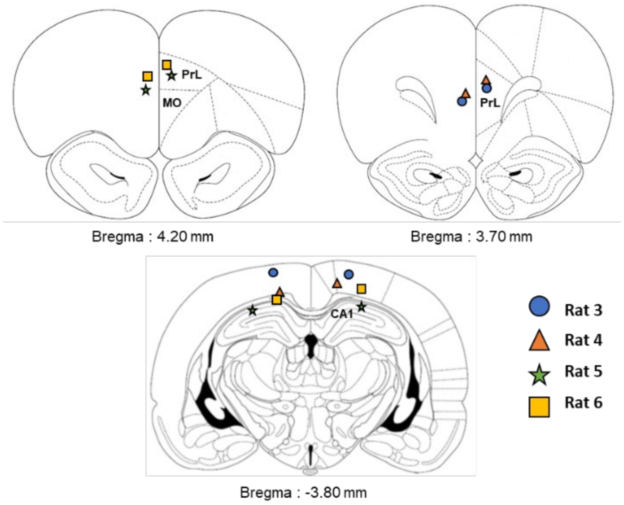

Fig. S2: Placement of electrodes after histological confirmation, STAR method bilateral wire drive implant.
Rat 3 did not reach the hippocampus and was only included for analysis of sleep stages and cortical events. All the other rats had at least one electrode with ripples.

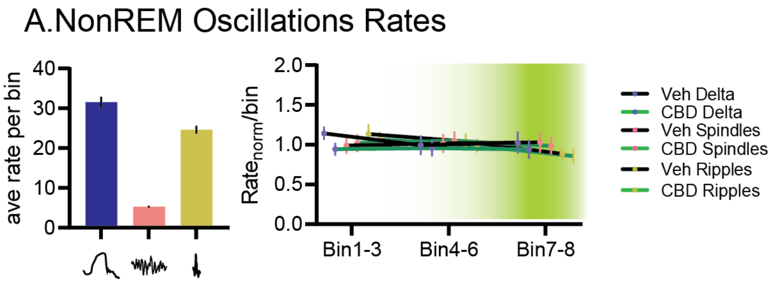

Fig. S3: Rates of NonREM oscillations, related to Fig. 3

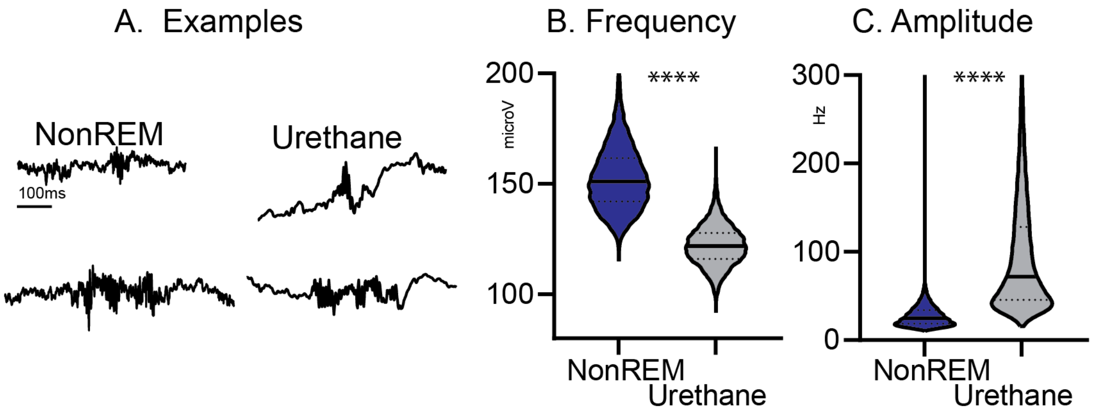

Fig. S4: NonREM and Urethane ripples, related to Fig. 3. Urethane came with slower and larger ripples. **** $p<0.0001$ K-S, Fig. 3
